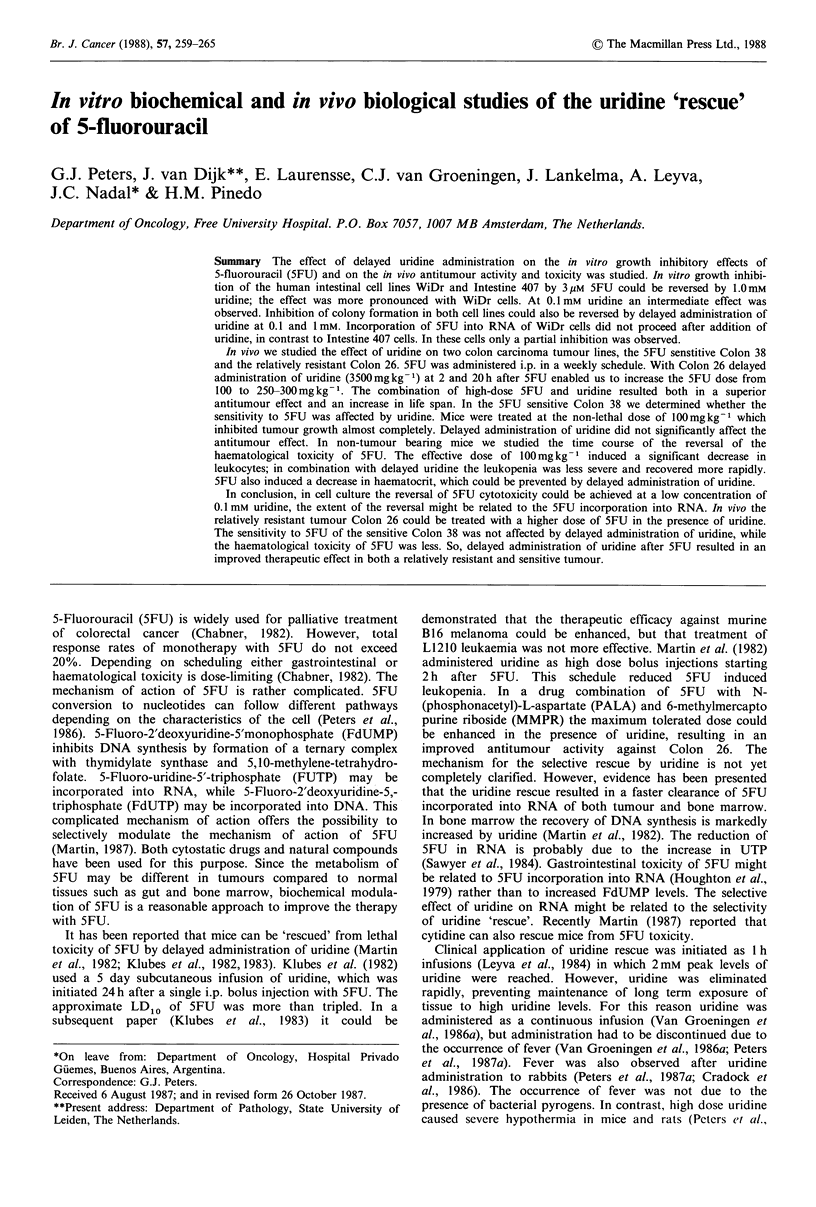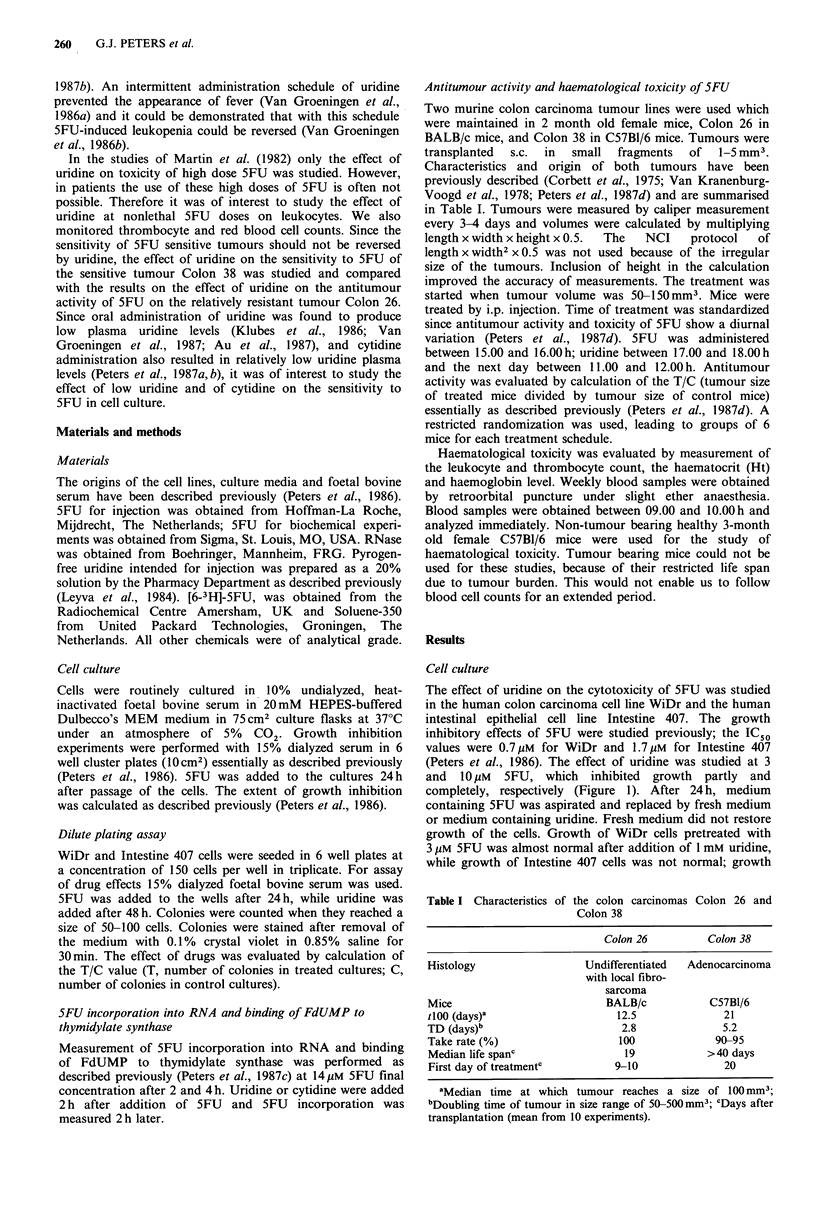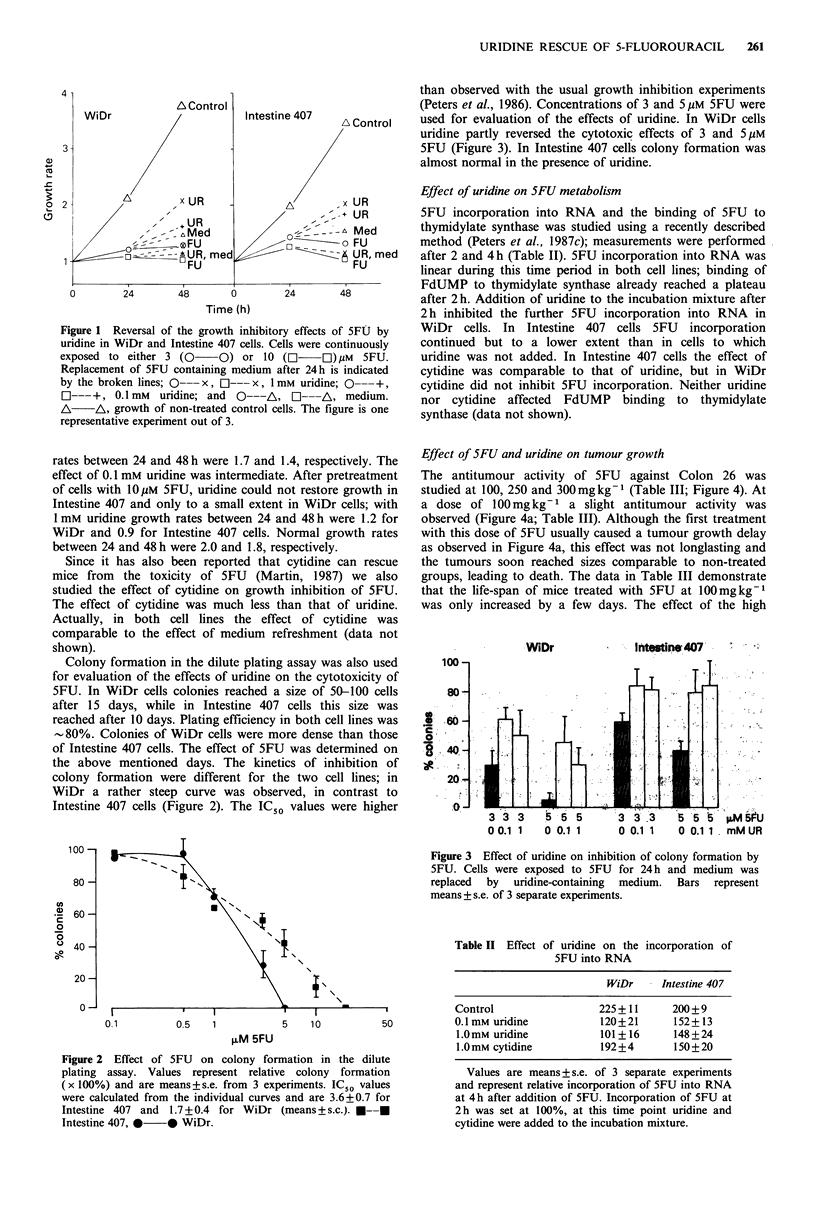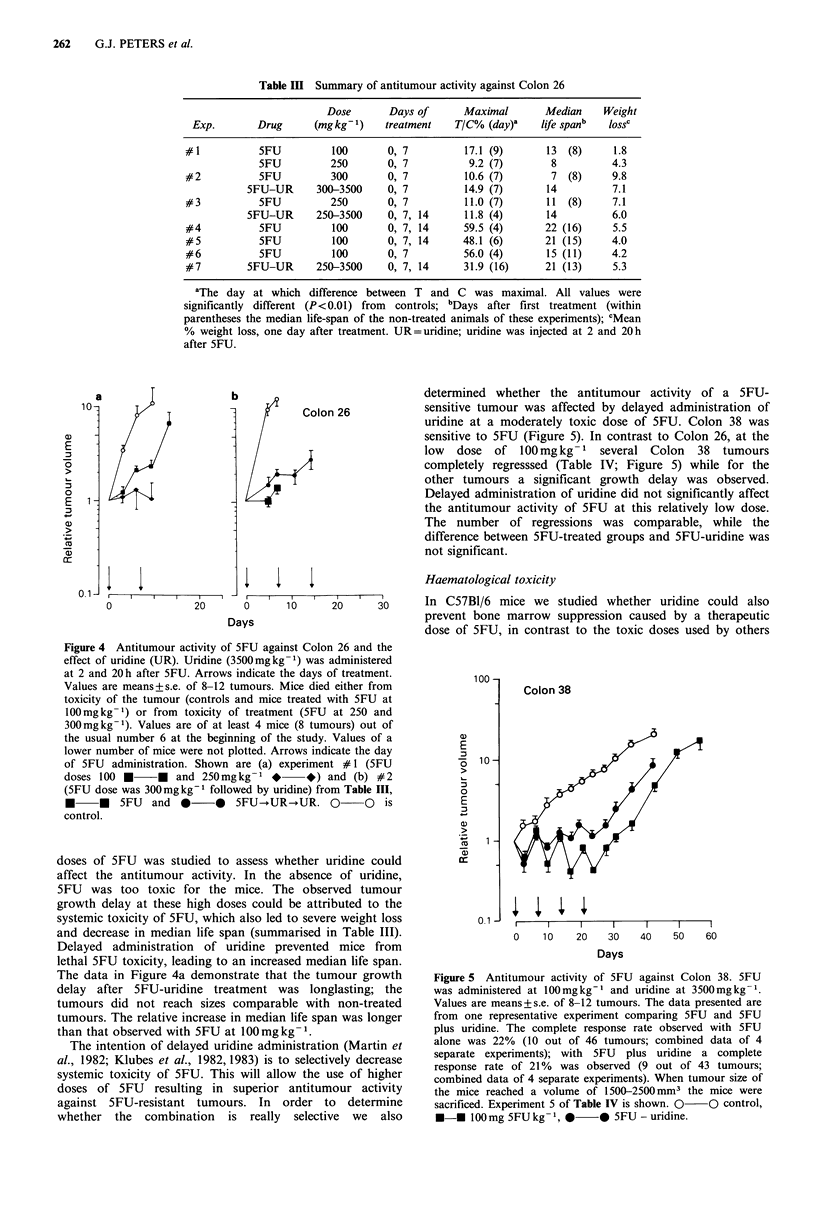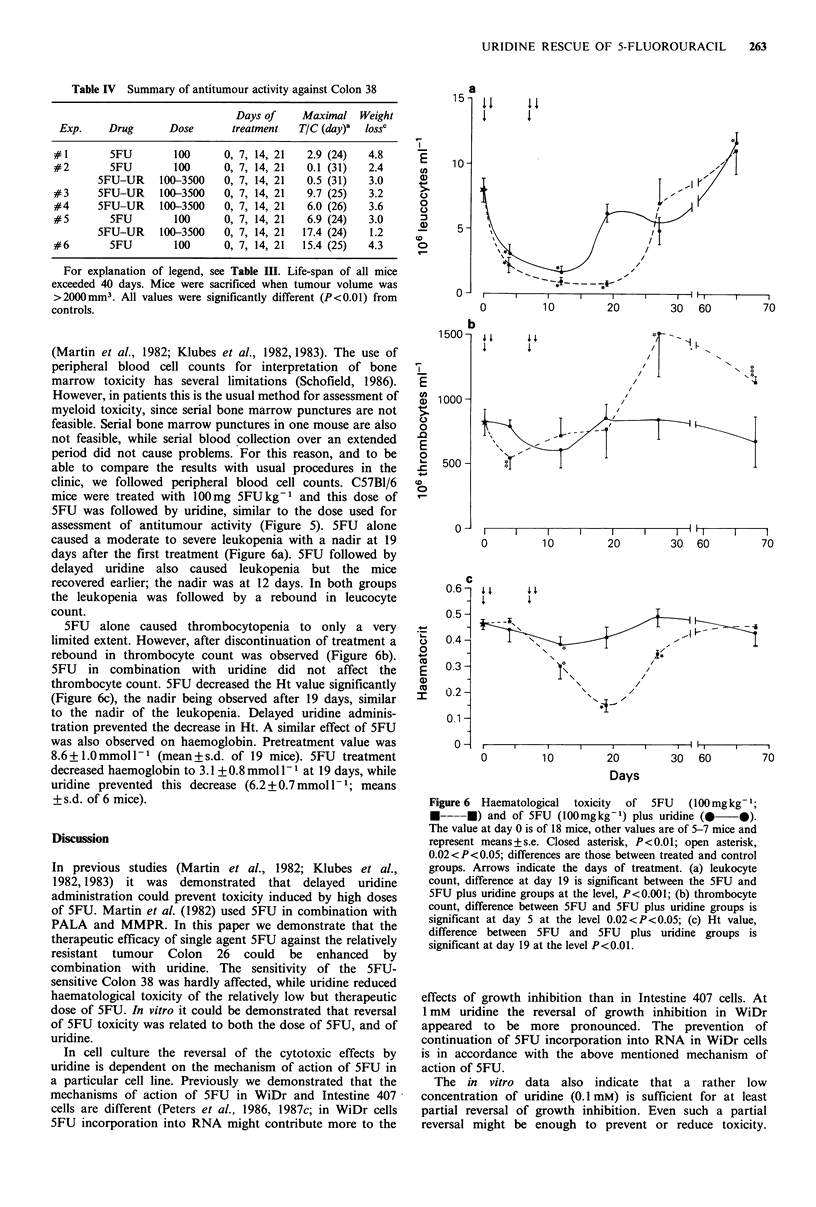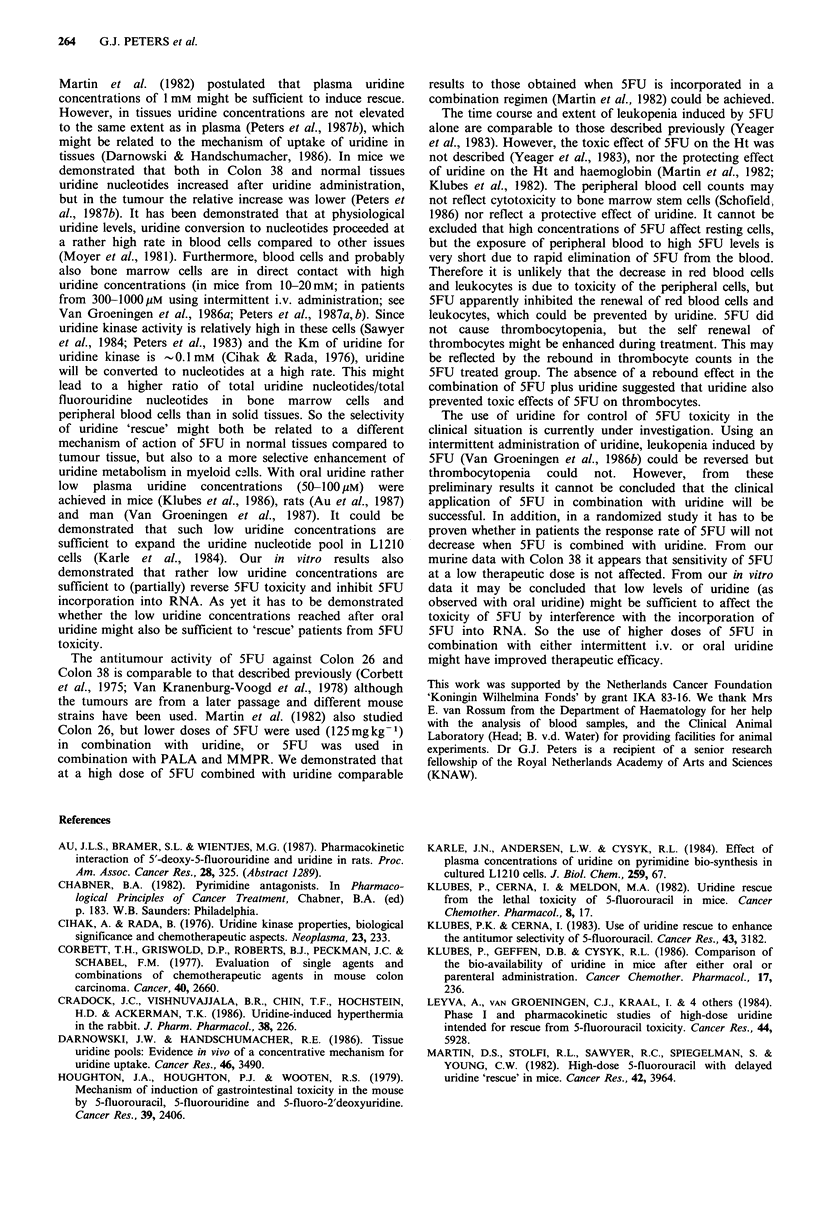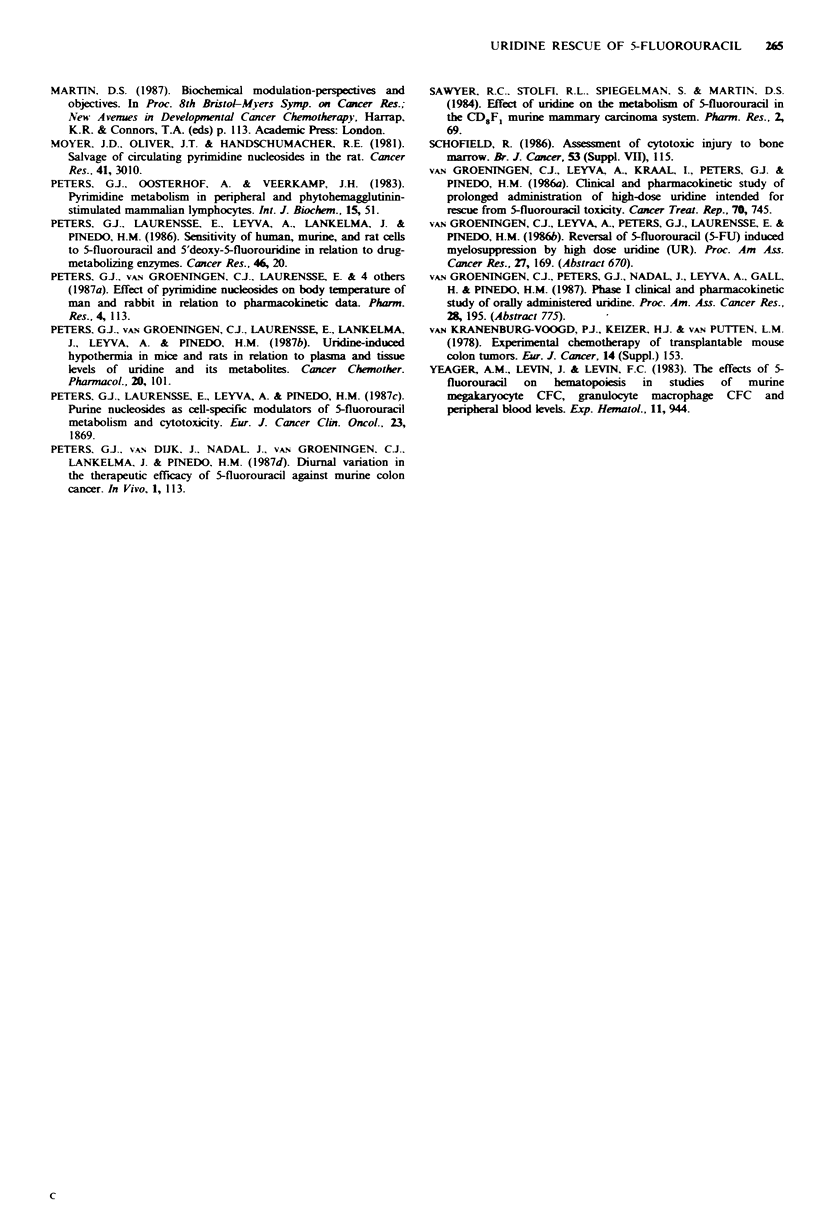# In vitro biochemical and in vivo biological studies of the uridine 'rescue' of 5-fluorouracil.

**DOI:** 10.1038/bjc.1988.56

**Published:** 1988-03

**Authors:** G. J. Peters, J. van Dijk, E. Laurensse, C. J. van Groeningen, J. Lankelma, A. Leyva, J. C. Nadal, H. M. Pinedo

**Affiliations:** Department of Oncology, Free University Hospital, Amsterdam, The Netherlands.

## Abstract

The effect of delayed uridine administration on the in vitro growth inhibitory effects of 5-fluorouracil (5FU) and on the in vivo antitumour activity and toxicity was studied. In vitro growth inhibition of the human intestinal cell lines WiDr and Intestine 407 by 3 microM 5FU could be reversed by 1.0 mM uridine; the effect was more pronounced with WiDr cells. At 0.1 mM uridine an intermediate effect was observed. Inhibition of colony formation in both cell lines could also be reversed by delayed administration of uridine at 0.1 and 1 mM. Incorporation of 5FU into RNA of WiDr cells did not proceed after addition of uridine, in contrast to Intestine 407 cells. In these cells only a partial inhibition was observed. In vivo we studied the effect of uridine on two colon carcinoma tumour lines, the 5FU sensitive Colon 38 and the relatively resistant Colon 26. 5FU was administered i.p. in a weekly schedule. With Colon 26 delayed administration of uridine (3500 mg kg-1) at 2 and 20 h after 5FU enabled us to increase the 5FU dose from 100 to 250 300mg kg-1. The combination of high-dose 5FU and uridine resulted both in a superior antitumour effect and an increase in life span. In the 5FU sensitive Colon 38 we determined whether the sensitivity to 5FU was affected by uridine. Mice were treated at the non-lethal dose of 100 mg kg-1 which inhibited tumour growth almost completely. Delayed administration of uridine did not significantly affect the antitumour effect. In non-tumour bearing mice we studied the time course of the reversal of the haematological toxicity of 5FU. The effective dose of 100 mg kg-1 induced a significant decrease in leukocytes; in combination with delayed uridine the leukopenia was less severe and recovered more rapidly. 5FU also induced a decrease in haematocrit, which could be prevented by delayed administration of uridine. In conclusion, in cell culture the reversal of 5FU cytotoxicity could be achieved at a low concentration of 0.1 mM uridine, the extent of the reversal might be related to the 5FU incorporation into RNA. In vivo the relatively resistant tumour Colon 26 could be treated with a higher dose of 5FU in the presence of uridine. The sensitivity to 5FU of the sensitive Colon 38 was not affected by delayed administration of uridine, while the haematological toxicity of 5FU was less. So, delayed administration of uridine after 5FU resulted in an improved therapeutic effect in both a relatively resistant and sensitive tumour.


Br. J. Cancer (1988), 57, 259–265 © The Macmillan Press Ltd., 1988

### *In vitro* biochemical and *in vivo* biological studies of the uridine 'rescue' of 5-fluorouracil

G.J. Peters, J. van Dijk**, E. Laurensse, C.J. van Groeningen, J. Lankelma, A. Leyva, J.C. Nadal* & H.M. Pinedo

*Department of Oncology, Free University Hospital. P.O. Box 7057, 1007 MB Amsterdam, The Netherlands.*

**Summary** The effect of delayed uridine administration on the *in vitro* growth inhibitory effects of 5-fluorouracil (5FU) and on the *in vivo* antitumour activity and toxicity was studied. *In vitro* growth inhibition of the human intestinal cell lines WiDr and Intestine 407 by 3 μM 5FU could be reversed by 1.0 mM uridine; the effect was more pronounced with WiDr cells. At 0.1 mM uridine an intermediate effect was observed. Inhibition of colony formation in both cell lines could also be reversed by delayed administration of uridine at 0.1 and 1 mM. Incorporation of 5FU into RNA of WiDr cells did not proceed after addition of uridine, in contrast to Intestine 407 cells. In these cells only a partial inhibition was observed.

*In vivo* we studied the effect of uridine on two colon carcinoma tumour lines, the 5FU senstitive Colon 38 and the relatively resistant Colon 26. 5FU was administered i.p. in a weekly schedule. With Colon 26 delayed administration of uridine (3500 mg kg$^{-1}$) at 2 and 20 h after 5FU enabled us to increase the 5FU dose from 100 to 250–300 mg kg$^{-1}$. The combination of high-dose 5FU and uridine resulted both in a superior antitumour effect and an increase in life span. In the 5FU sensitive Colon 38 we determined whether the sensitivity to 5FU was affected by uridine. Mice were treated at the non-lethal dose of 100 mg kg$^{-1}$ which inhibited tumour growth almost completely. Delayed administration of uridine did not significantly affect the antitumour effect. In non-tumour bearing mice we studied the time course of the reversal of the haematological toxicity of 5FU. The effective dose of 100 mg kg$^{-1}$ induced a significant decrease in leukocytes; in combination with delayed uridine the leukopenia was less severe and recovered more rapidly. 5FU also induced a decrease in haematocrit, which could be prevented by delayed administration of uridine.

In conclusion, in cell culture the reversal of 5FU cytotoxicity could be achieved at a low concentration of 0.1 mM uridine, the extent of the reversal might be related to the 5FU incorporation into RNA. *In vivo* the relatively resistant tumour Colon 26 could be treated with a higher dose of 5FU in the presence of uridine. The sensitivity to 5FU of the sensitive Colon 38 was not affected by delayed administration of uridine, while the haematological toxicity of 5FU was less. So, delayed administration of uridine after 5FU resulted in an improved therapeutic effect in both a relatively resistant and sensitive tumour.

5-Fluorouracil (5FU) is widely used for palliative treatment of colorectal cancer (Chabner, 1982). However, total response rates of monotherapy with 5FU do not exceed 20%. Depending on scheduling either gastrointestinal or haematological toxicity is dose-limiting (Chabner, 1982). The mechanism of action of 5FU is rather complicated. 5FU conversion to nucleotides can follow different pathways depending on the characteristics of the cell (Peters *et al.*, 1986). 5-Fluoro-2′deoxyuridine-5′monophosphate (FdUMP) inhibits DNA synthesis by formation of a ternary complex with thymidylate synthase and 5,10-methylene-tetrahydrofolate. 5-Fluoro-uridine-5′-triphosphate (FUTP) may be incorporated into RNA, while 5-Fluoro-2′deoxyuridine-5,-triphosphate (FdUTP) may be incorporated into DNA. This complicated mechanism of action offers the possibility to selectively modulate the mechanism of action of 5FU (Martin, 1987). Both cytostatic drugs and natural compounds have been used for this purpose. Since the metabolism of 5FU may be different in tumours compared to normal tissues such as gut and bone marrow, biochemical modulation of 5FU is a reasonable approach to improve the therapy with 5FU.

It has been reported that mice can be 'rescued' from lethal toxicity of 5FU by delayed administration of uridine (Martin *et al.*, 1982; Klubes *et al.*, 1982, 1983). Klubes *et al.* (1982) used a 5 day subcutaneous infusion of uridine, which was initiated 24 h after a single i.p. bolus injection with 5FU. The approximate $LD_{10}$ of 5FU was more than tripled. In a subsequent paper (Klubes *et al.*, 1983) it could be demonstrated that the therapeutic efficacy against murine B16 melanoma could be enhanced, but that treatment of L1210 leukaemia was not more effective. Martin *et al.* (1982) administered uridine as high dose bolus injections starting 2 h after 5FU. This schedule reduced 5FU induced leukopenia. In a drug combination of 5FU with N-(phosphonacetyl)-L-aspartate (PALA) and 6-methylmercapto purine riboside (MMPR) the maximum tolerated dose could be enhanced in the presence of uridine, resulting in an improved antitumour activity against Colon 26. The mechanism for the selective rescue by uridine is not yet completely clarified. However, evidence has been presented that the uridine rescue resulted in a faster clearance of 5FU incorporated into RNA of both tumour and bone marrow. In bone marrow the recovery of DNA synthesis is markedly increased by uridine (Martin *et al.*, 1982). The reduction of 5FU in RNA is probably due to the increase in UTP (Sawyer *et al.*, 1984). Gastrointestinal toxicity of 5FU might be related to 5FU incorporation into RNA (Houghton *et al.*, 1979) rather than to increased FdUMP levels. The selective effect of uridine on RNA might be related to the selectivity of uridine 'rescue'. Recently Martin (1987) reported that cytidine can also rescue mice from 5FU toxicity.

Clinical application of uridine rescue was initiated as 1 h infusions (Leyva *et al.*, 1984) in which 2 mM peak levels of uridine were reached. However, uridine was eliminated rapidly, preventing maintenance of long term exposure of tissue to high uridine levels. For this reason uridine was administered as a continuous infusion (Van Groeningen *et al.*, 1986*a*), but administration had to be discontinued due to the occurrence of fever (Van Groeningen *et al.*, 1986*a*; Peters *et al.*, 1987*a*). Fever was also observed after uridine administration to rabbits (Peters *et al.*, 1987*a*; Cradock *et al.*, 1986). The occurrence of fever was not due to the presence of bacterial pyrogens. In contrast, high dose uridine caused severe hypothermia in mice and rats (Peters *et al.*,

*On leave from: Department of Oncology, Hospital Privado Güemes, Buenos Aires, Argentina.
Correspondence: G.J. Peters.
Received 6 August 1987; and in revised form 26 October 1987.
**Present address: Department of Pathology, State University of Leiden, The Netherlands.

1987b). An intermittent administration schedule of uridine prevented the appearance of fever (Van Groeningen et al., 1986a) and it could be demonstrated that with this schedule 5FU-induced leukopenia could be reversed (Van Groeningen et al., 1986b).

In the studies of Martin et al. (1982) only the effect of uridine on toxicity of high dose 5FU was studied. However, in patients the use of these high doses of 5FU is often not possible. Therefore it was of interest to study the effect of uridine at nonlethal 5FU doses on leukocytes. We also monitored thrombocyte and red blood cell counts. Since the sensitivity of 5FU sensitive tumours should not be reversed by uridine, the effect of uridine on the sensitivity to 5FU of the sensitive tumour Colon 38 was studied and compared with the results on the effect of uridine on the antitumour activity of 5FU on the relatively resistant tumour Colon 26. Since oral administration of uridine was found to produce low plasma uridine levels (Klubes et al., 1986; Van Groeningen et al., 1987; Au et al., 1987), and cytidine administration also resulted in relatively low uridine plasma levels (Peters et al., 1987a, b), it was of interest to study the effect of low uridine and of cytidine on the sensitivity to 5FU in cell culture.

### Materials and methods

### Materials

The origins of the cell lines, culture media and foetal bovine serum have been described previously (Peters et al., 1986). 5FU for injection was obtained from Hoffman-La Roche, Mijdrecht, The Netherlands; 5FU for biochemical experiments was obtained from Sigma, St. Louis, MO, USA. RNase was obtained from Boehringer, Mannheim, FRG. Pyrogen-free uridine intended for injection was prepared as a 20% solution by the Pharmacy Department as described previously (Leyva et al., 1984). [6-$^3$H]-5FU, was obtained from the Radiochemical Centre Amersham, UK and Soluene-350 from United Packard Technologies, Groningen, The Netherlands. All other chemicals were of analytical grade.

### Cell culture

Cells were routinely cultured in 10% undialyzed, heat-inactivated foetal bovine serum in 20 mM HEPES-buffered Dulbecco's MEM medium in 75 cm$^2$ culture flasks at 37°C under an atmosphere of 5% $CO_2$. Growth inhibition experiments were performed with 15% dialyzed serum in 6 well cluster plates (10 cm$^2$) essentially as described previously (Peters et al., 1986). 5FU was added to the cultures 24 h after passage of the cells. The extent of growth inhibition was calculated as described previously (Peters et al., 1986).

### Dilute plating assay

WiDr and Intestine 407 cells were seeded in 6 well plates at a concentration of 150 cells per well in triplicate. For assay of drug effects 15% dialyzed foetal bovine serum was used. 5FU was added to the wells after 24 h, while uridine was added after 48 h. Colonies were counted when they reached a size of 50–100 cells. Colonies were stained after removal of the medium with 0.1% crystal violet in 0.85% saline for 30 min. The effect of drugs was evaluated by calculation of the T/C value (T, number of colonies in treated cultures; C, number of colonies in control cultures).

### 5FU incorporation into RNA and binding of FdUMP to thymidylate synthase

Measurement of 5FU incorporation into RNA and binding of FdUMP to thymidylate synthase was performed as described previously (Peters et al., 1987c) at 14 $\mu$M 5FU final concentration after 2 and 4 h. Uridine or cytidine were added 2 h after addition of 5FU and 5FU incorporation was measured 2 h later.

### Antitumour activity and haematological toxicity of 5FU

Two murine colon carcinoma tumour lines were used which were maintained in 2 month old female mice, Colon 26 in BALB/c mice, and Colon 38 in C57Bl/6 mice. Tumours were transplanted s.c. in small fragments of 1–5 mm$^3$. Characteristics and origin of both tumours have been previously described (Corbett et al., 1975; Van Kranenburg-Voogd et al., 1978; Peters et al., 1987d) and are summarised in Table I. Tumours were measured by caliper measurement every 3–4 days and volumes were calculated by multiplying length × width × height × 0.5. The NCI protocol of length × width$^2$ × 0.5 was not used because of the irregular size of the tumours. Inclusion of height in the calculation improved the accuracy of measurements. The treatment was started when tumour volume was 50–150 mm$^3$. Mice were treated by i.p. injection. Time of treatment was standardized since antitumour activity and toxicity of 5FU show a diurnal variation (Peters et al., 1987d). 5FU was administered between 15.00 and 16.00 h; uridine between 17.00 and 18.00 h and the next day between 11.00 and 12.00 h. Antitumour activity was evaluated by calculation of the T/C (tumour size of treated mice divided by tumour size of control mice) essentially as described previously (Peters et al., 1987d). A restricted randomization was used, leading to groups of 6 mice for each treatment schedule.

Haematological toxicity was evaluated by measurement of the leukocyte and thrombocyte count, the haematocrit (Ht) and haemoglobin level. Weekly blood samples were obtained by retroorbital puncture under slight ether anaesthesia. Blood samples were obtained between 09.00 and 10.00 h and analyzed immediately. Non-tumour bearing healthy 3-month old female C57Bl/6 mice were used for the study of haematological toxicity. Tumour bearing mice could not be used for these studies, because of their restricted life span due to tumour burden. This would not enable us to follow blood cell counts for an extended period.

### Results

### Cell culture

The effect of uridine on the cytotoxicity of 5FU was studied in the human colon carcinoma cell line WiDr and the human intestinal epithelial cell line Intestine 407. The growth inhibitory effects of 5FU were studied previously; the $IC_{50}$ values were 0.7 $\mu$M for WiDr and 1.7 $\mu$M for Intestine 407 (Peters et al., 1986). The effect of uridine was studied at 3 and 10 $\mu$M 5FU, which inhibited growth partly and completely, respectively (Figure 1). After 24 h, medium containing 5FU was aspirated and replaced by fresh medium or medium containing uridine. Fresh medium did not restore growth of the cells. Growth of WiDr cells pretreated with 3 $\mu$M 5FU was almost normal after addition of 1 mM uridine, while growth of Intestine 407 cells was not normal; growth

**Table I** Characteristics of the colon carcinomas Colon 26 and Colon 38

| | Colon 26 | Colon 38 |
|---|---|---|
| Histology | Undifferentiated with local fibrosarcoma | Adenocarcinoma |
| Mice | BALB/c | C57Bl/6 |
| t100 (days)[a] | 12.5 | 21 |
| TD (days)[b] | 2.8 | 5.2 |
| Take rate (%) | 100 | 90–95 |
| Median life span[c] | 19 | >40 days |
| First day of treatment[c] | 9–10 | 20 |

[a]Median time at which tumour reaches a size of 100 mm$^3$; [b]Doubling time of tumour in size range of 50–500 mm$^3$; [c]Days after transplantation (mean from 10 experiments).

**Figure 1** Reversal of the growth inhibitory effects of 5FU by uridine in WiDr and Intestine 407 cells. Cells were continuously exposed to either 3 (○——○) or 10 (□——□) μM 5FU. Replacement of 5FU containing medium after 24 h is indicated by the broken lines; ○---×, □---×, 1 mM uridine; ○---+, □---+, 0.1 mM uridine; and ○---△, □---△, medium. △——△, growth of non-treated control cells. The figure is one representative experiment out of 3.

rates between 24 and 48 h were 1.7 and 1.4, respectively. The effect of 0.1 mM uridine was intermediate. After pretreatment of cells with 10 μM 5FU, uridine could not restore growth in Intestine 407 and only to a small extent in WiDr cells; with 1 mM uridine growth rates between 24 and 48 h were 1.2 for WiDr and 0.9 for Intestine 407 cells. Normal growth rates between 24 and 48 h were 2.0 and 1.8, respectively.

Since it has also been reported that cytidine can rescue mice from the toxicity of 5FU (Martin, 1987) we also studied the effect of cytidine on growth inhibition of 5FU. The effect of cytidine was much less than that of uridine. Actually, in both cell lines the effect of cytidine was comparable to the effect of medium refreshment (data not shown).

Colony formation in the dilute plating assay was also used for evaluation of the effects of uridine on the cytotoxicity of 5FU. In WiDr cells colonies reached a size of 50–100 cells after 15 days, while in Intestine 407 cells this size was reached after 10 days. Plating efficiency in both cell lines was ~80%. Colonies of WiDr cells were more dense than those of Intestine 407 cells. The effect of 5FU was determined on the above mentioned days. The kinetics of inhibition of colony formation were different for the two cell lines; in WiDr a rather steep curve was observed, in contrast to Intestine 407 cells (Figure 2). The $IC_{50}$ values were higher

**Figure 2** Effect of 5FU on colony formation in the dilute plating assay. Values represent relative colony formation (×100%) and are means ± s.e. from 3 experiments. $IC_{50}$ values were calculated from the individual curves and are 3.6 ± 0.7 for Intestine 407 and 1.7 ± 0.4 for WiDr (means ± s.c.). ■--■ Intestine 407, ●——● WiDr.

than observed with the usual growth inhibition experiments (Peters *et al.*, 1986). Concentrations of 3 and 5 μM 5FU were used for evaluation of the effects of uridine. In WiDr cells uridine partly reversed the cytotoxic effects of 3 and 5 μM 5FU (Figure 3). In Intestine 407 cells colony formation was almost normal in the presence of uridine.

*Effect of uridine on 5FU metabolism*

5FU incorporation into RNA and the binding of 5FU to thymidylate synthase was studied using a recently described method (Peters *et al.*, 1987*c*); measurements were performed after 2 and 4 h (Table II). 5FU incorporation into RNA was linear during this time period in both cell lines; binding of FdUMP to thymidylate synthase already reached a plateau after 2 h. Addition of uridine to the incubation mixture after 2 h inhibited the further 5FU incorporation into RNA in WiDr cells. In Intestine 407 cells 5FU incorporation continued but to a lower extent than in cells to which uridine was not added. In Intestine 407 cells the effect of cytidine was comparable to that of uridine, but in WiDr cytidine did not inhibit 5FU incorporation. Neither uridine nor cytidine affected FdUMP binding to thymidylate synthase (data not shown).

*Effect of 5FU and uridine on tumour growth*

The antitumour activity of 5FU against Colon 26 was studied at 100, 250 and 300 mg kg$^{-1}$ (Table III; Figure 4). At a dose of 100 mg kg$^{-1}$ a slight antitumour activity was observed (Figure 4a; Table III). Although the first treatment with this dose of 5FU usually caused a tumour growth delay as observed in Figure 4a, this effect was not longlasting and the tumours soon reached sizes comparable to non-treated groups, leading to death. The data in Table III demonstrate that the life-span of mice treated with 5FU at 100 mg kg$^{-1}$ was only increased by a few days. The effect of the high

**Figure 3** Effect of uridine on inhibition of colony formation by 5FU. Cells were exposed to 5FU for 24 h and medium was replaced by uridine-containing medium. Bars represent means ± s.e. of 3 separate experiments.

**Table II** Effect of uridine on the incorporation of 5FU into RNA

| | *WiDr* | *Intestine 407* |
|---|---|---|
| Control | 225 ± 11 | 200 ± 9 |
| 0.1 mM uridine | 120 ± 21 | 152 ± 13 |
| 1.0 mM uridine | 101 ± 16 | 148 ± 24 |
| 1.0 mM cytidine | 192 ± 4 | 150 ± 20 |

Values are means ± s.e. of 3 separate experiments and represent relative incorporation of 5FU into RNA at 4 h after addition of 5FU. Incorporation of 5FU at 2 h was set at 100%, at this time point uridine and cytidine were added to the incubation mixture.

Table III Summary of antitumour activity against Colon 26

| Exp. | Drug | Dose ($mg\,kg^{-1}$) | Days of treatment | Maximal T/C% (day)[a] | Median life span[b] | Weight loss[c] |
|---|---|---|---|---|---|---|
| #1 | 5FU | 100 | 0, 7 | 17.1 (9) | 13 (8) | 1.8 |
| | 5FU | 250 | 0, 7 | 9.2 (7) | 8 | 4.3 |
| #2 | 5FU | 300 | 0, 7 | 10.6 (7) | 7 (8) | 9.8 |
| | 5FU-UR | 300–3500 | 0, 7 | 14.9 (7) | 14 | 7.1 |
| #3 | 5FU | 250 | 0, 7 | 11.0 (7) | 11 (8) | 7.1 |
| | 5FU-UR | 250–3500 | 0, 7, 14 | 11.8 (4) | 14 | 6.0 |
| #4 | 5FU | 100 | 0, 7, 14 | 59.5 (4) | 22 (16) | 5.5 |
| #5 | 5FU | 100 | 0, 7, 14 | 48.1 (6) | 21 (15) | 4.0 |
| #6 | 5FU | 100 | 0, 7 | 56.0 (4) | 15 (11) | 4.2 |
| #7 | 5FU-UR | 250–3500 | 0, 7, 14 | 31.9 (16) | 21 (13) | 5.3 |

[a]The day at which difference between T and C was maximal. All values were significantly different ($P<0.01$) from controls; [b]Days after first treatment (within parentheses the median life-span of the non-treated animals of these experiments); [c]Mean % weight loss, one day after treatment. UR = uridine; uridine was injected at 2 and 20 h after 5FU.

Figure 4 Antitumour activity of 5FU against Colon 26 and the effect of uridine (UR). Uridine ($3500\,mg\,kg^{-1}$) was administered at 2 and 20 h after 5FU. Arrows indicate the days of treatment. Values are means ± s.e. of 8–12 tumours. Mice died either from toxicity of the tumour (controls and mice treated with 5FU at $100\,mg\,kg^{-1}$) or from toxicity of treatment (5FU at 250 and $300\,mg\,kg^{-1}$). Values are of at least 4 mice (8 tumours) out of the usual number 6 at the beginning of the study. Values of a lower number of mice were not plotted. Arrows indicate the day of 5FU administration. Shown are (a) experiment #1 (5FU doses 100 ■——■ and $250\,mg\,kg^{-1}$ ◆——◆) and (b) #2 (5FU dose was $300\,mg\,kg^{-1}$ followed by uridine) from **Table III**, ■——■ 5FU and ●——● 5FU→UR→UR. ○——○ is control.

doses of 5FU was studied to assess whether uridine could affect the antitumour activity. In the absence of uridine, 5FU was too toxic for the mice. The observed tumour growth delay at these high doses could be attributed to the systemic toxicity of 5FU, which also led to severe weight loss and decrease in median life span (summarised in Table III). Delayed administration of uridine prevented mice from lethal 5FU toxicity, leading to an increased median life span. The data in Figure 4a demonstrate that the tumour growth delay after 5FU-uridine treatment was longlasting; the tumours did not reach sizes comparable with non-treated tumours. The relative increase in median life span was longer than that observed with 5FU at $100\,mg\,kg^{-1}$.

The intention of delayed uridine administration (Martin *et al.*, 1982; Klubes *et al.*, 1982, 1983) is to selectively decrease systemic toxicity of 5FU. This will allow the use of higher doses of 5FU resulting in superior antitumour activity against 5FU-resistant tumours. In order to determine whether the combination is really selective we also determined whether the antitumour activity of a 5FU-sensitive tumour was affected by delayed administration of uridine at a moderately toxic dose of 5FU. Colon 38 was sensitive to 5FU (Figure 5). In contrast to Colon 26, at the low dose of $100\,mg\,kg^{-1}$ several Colon 38 tumours completely regresssed (Table IV; Figure 5) while for the other tumours a significant growth delay was observed. Delayed administration of uridine did not significantly affect the antitumour activity of 5FU at this relatively low dose. The number of regressions was comparable, while the difference between 5FU-treated groups and 5FU-uridine was not significant.

*Haematological toxicity*

In C57Bl/6 mice we studied whether uridine could also prevent bone marrow suppression caused by a therapeutic dose of 5FU, in contrast to the toxic doses used by others

Figure 5 Antitumour activity of 5FU against Colon 38. 5FU was administered at $100\,mg\,kg^{-1}$ and uridine at $3500\,mg\,kg^{-1}$. Values are means ± s.e. of 8–12 tumours. The data presented are from one representative experiment comparing 5FU and 5FU plus uridine. The complete response rate observed with 5FU alone was 22% (10 out of 46 tumours; combined data of 4 separate experiments); with 5FU plus uridine a complete response rate of 21% was observed (9 out of 43 tumours; combined data of 4 separate experiments). When tumour size of the mice reached a volume of $1500–2500\,mm^3$ the mice were sacrificed. Experiment 5 of **Table IV** is shown. ○——○ control, ■—■ 100 mg 5FU $kg^{-1}$, ●——● 5FU – uridine.

**Table IV** Summary of antitumour activity against Colon 38

| *Exp.* | *Drug* | *Dose* | *Days of treatment* | *Maximal T/C (day)*[a] | *Weight loss*[c] |
|---|---|---|---|---|---|
| #1 | 5FU | 100 | 0, 7, 14, 21 | 2.9 (24) | 4.8 |
| #2 | 5FU | 100 | 0, 7, 14, 21 | 0.1 (31) | 2.4 |
| | 5FU-UR | 100–3500 | 0, 7, 14, 21 | 0.5 (31) | 3.0 |
| #3 | 5FU-UR | 100–3500 | 0, 7, 14, 21 | 9.7 (25) | 3.2 |
| #4 | 5FU-UR | 100–3500 | 0, 7, 14, 21 | 6.0 (26) | 3.6 |
| #5 | 5FU | 100 | 0, 7, 14, 21 | 6.9 (24) | 3.0 |
| | 5FU-UR | 100–3500 | 0, 7, 14, 21 | 17.4 (24) | 1.2 |
| #6 | 5FU | 100 | 0, 7, 14, 21 | 15.4 (25) | 4.3 |

For explanation of legend, see **Table III**. Life-span of all mice exceeded 40 days. Mice were sacrificed when tumour volume was $>2000\,mm^3$. All values were significantly different ($P<0.01$) from controls.

(Martin *et al.*, 1982; Klubes *et al.*, 1982, 1983). The use of peripheral blood cell counts for interpretation of bone marrow toxicity has several limitations (Schofield, 1986). However, in patients this is the usual method for assessment of myeloid toxicity, since serial bone marrow punctures are not feasible. Serial bone marrow punctures in one mouse are also not feasible, while serial blood collection over an extended period did not cause problems. For this reason, and to be able to compare the results with usual procedures in the clinic, we followed peripheral blood cell counts. C57Bl/6 mice were treated with 100 mg $5FU\,kg^{-1}$ and this dose of 5FU was followed by uridine, similar to the dose used for assessment of antitumour activity (Figure 5). 5FU alone caused a moderate to severe leukopenia with a nadir at 19 days after the first treatment (Figure 6a). 5FU followed by delayed uridine also caused leukopenia but the mice recovered earlier; the nadir was at 12 days. In both groups the leukopenia was followed by a rebound in leucocyte count.

5FU alone caused thrombocytopenia to only a very limited extent. However, after discontinuation of treatment a rebound in thrombocyte count was observed (Figure 6b). 5FU in combination with uridine did not affect the thrombocyte count. 5FU decreased the Ht value significantly (Figure 6c), the nadir being observed after 19 days, similar to the nadir of the leukopenia. Delayed uridine administration prevented the decrease in Ht. A similar effect of 5FU was also observed on haemoglobin. Pretreatment value was $8.6\pm1.0\,mmol\,l^{-1}$ (mean±s.d. of 19 mice). 5FU treatment decreased haemoglobin to $3.1\pm0.8\,mmol\,l^{-1}$ at 19 days, while uridine prevented this decrease ($6.2\pm0.7\,mmol\,l^{-1}$; means ±s.d. of 6 mice).

**Figure 6** Haematological toxicity of 5FU ($100\,mg\,kg^{-1}$; ■----■) and of 5FU ($100\,mg\,kg^{-1}$) plus uridine (●——●). The value at day 0 is of 18 mice, other values are of 5–7 mice and represent means±s.e. Closed asterisk, $P<0.01$; open asterisk, $0.02<P<0.05$; differences are those between treated and control groups. Arrows indicate the days of treatment. (a) leukocyte count, difference at day 19 is significant between the 5FU and 5FU plus uridine groups at the level, $P<0.001$; (b) thrombocyte count, difference between 5FU and 5FU plus uridine groups is significant at day 5 at the level $0.02<P<0.05$; (c) Ht value, difference between 5FU and 5FU plus uridine groups is significant at day 19 at the level $P<0.01$.

### Discussion

In previous studies (Martin *et al.*, 1982; Klubes *et al.*, 1982, 1983) it was demonstrated that delayed uridine administration could prevent toxicity induced by high doses of 5FU. Martin *et al.* (1982) used 5FU in combination with PALA and MMPR. In this paper we demonstrate that the therapeutic efficacy of single agent 5FU against the relatively resistant tumour Colon 26 could be enhanced by combination with uridine. The sensitivity of the 5FU-sensitive Colon 38 was hardly affected, while uridine reduced haematological toxicity of the relatively low but therapeutic dose of 5FU. *In vitro* it could be demonstrated that reversal of 5FU toxicity was related to both the dose of 5FU, and of uridine.

In cell culture the reversal of the cytotoxic effects by uridine is dependent on the mechanism of action of 5FU in a particular cell line. Previously we demonstrated that the mechanisms of action of 5FU in WiDr and Intestine 407 cells are different (Peters *et al.*, 1986, 1987*c*; in WiDr cells 5FU incorporation into RNA might contribute more to the effects of growth inhibition than in Intestine 407 cells. At 1 mM uridine the reversal of growth inhibition in WiDr appeared to be more pronounced. The prevention of continuation of 5FU incorporation into RNA in WiDr cells is in accordance with the above mentioned mechanism of action of 5FU.

The *in vitro* data also indicate that a rather low concentration of uridine (0.1 mM) is sufficient for at least partial reversal of growth inhibition. Even such a partial reversal might be enough to prevent or reduce toxicity.

Martin *et al.* (1982) postulated that plasma uridine concentrations of 1 mM might be sufficient to induce rescue. However, in tissues uridine concentrations are not elevated to the same extent as in plasma (Peters *et al.*, 1987*b*), which might be related to the mechanism of uptake of uridine in tissues (Darnowski & Handschumacher, 1986). In mice we demonstrated that both in Colon 38 and normal tissues uridine nucleotides increased after uridine administration, but in the tumour the relative increase was lower (Peters *et al.*, 1987*b*). It has been demonstrated that at physiological uridine levels, uridine conversion to nucleotides proceeded at a rather high rate in blood cells compared to other issues (Moyer *et al.*, 1981). Furthermore, blood cells and probably also bone marrow cells are in direct contact with high uridine concentrations (in mice from 10–20 mM; in patients from 300–1000 $\mu$M using intermittent i.v. administration; see Van Groeningen *et al.*, 1986*a*; Peters *et al.*, 1987*a*,*b*). Since uridine kinase activity is relatively high in these cells (Sawyer *et al.*, 1984; Peters *et al.*, 1983) and the Km of uridine for uridine kinase is ~0.1 mM (Cihak & Rada, 1976), uridine will be converted to nucleotides at a high rate. This might lead to a higher ratio of total uridine nucleotides/total fluorouridine nucleotides in bone marrow cells and peripheral blood cells than in solid tissues. So the selectivity of uridine 'rescue' might both be related to a different mechanism of action of 5FU in normal tissues compared to tumour tissue, but also to a more selective enhancement of uridine metabolism in myeloid cells. With oral uridine rather low plasma uridine concentrations (50–100 $\mu$M) were achieved in mice (Klubes *et al.*, 1986), rats (Au *et al.*, 1987) and man (Van Groeningen *et al.*, 1987). It could be demonstrated that such low uridine concentrations are sufficient to expand the uridine nucleotide pool in L1210 cells (Karle *et al.*, 1984). Our *in vitro* results also demonstrated that rather low uridine concentrations are sufficient to (partially) reverse 5FU toxicity and inhibit 5FU incorporation into RNA. As yet it has to be demonstrated whether the low uridine concentrations reached after oral uridine might also be sufficient to 'rescue' patients from 5FU toxicity.

The antitumour activity of 5FU against Colon 26 and Colon 38 is comparable to that described previously (Corbett *et al.*, 1975; Van Kranenburg-Voogd *et al.*, 1978) although the tumours are from a later passage and different mouse strains have been used. Martin *et al.* (1982) also studied Colon 26, but lower doses of 5FU were used (125 mg kg$^{-1}$) in combination with uridine, or 5FU was used in combination with PALA and MMPR. We demonstrated that at a high dose of 5FU combined with uridine comparable results to those obtained when 5FU is incorporated in a combination regimen (Martin *et al.*, 1982) could be achieved.

The time course and extent of leukopenia induced by 5FU alone are comparable to those described previously (Yeager *et al.*, 1983). However, the toxic effect of 5FU on the Ht was not described (Yeager *et al.*, 1983), nor the protecting effect of uridine on the Ht and haemoglobin (Martin *et al.*, 1982; Klubes *et al.*, 1982). The peripheral blood cell counts may not reflect cytotoxicity to bone marrow stem cells (Schofield, 1986) nor reflect a protective effect of uridine. It cannot be excluded that high concentrations of 5FU affect resting cells, but the exposure of peripheral blood to high 5FU levels is very short due to rapid elimination of 5FU from the blood. Therefore it is unlikely that the decrease in red blood cells and leukocytes is due to toxicity of the peripheral cells, but 5FU apparently inhibited the renewal of red blood cells and leukocytes, which could be prevented by uridine. 5FU did not cause thrombocytopenia, but the self renewal of thrombocytes might be enhanced during treatment. This may be reflected by the rebound in thrombocyte counts in the 5FU treated group. The absence of a rebound effect in the combination of 5FU plus uridine suggested that uridine also prevented toxic effects of 5FU on thrombocytes.

The use of uridine for control of 5FU toxicity in the clinical situation is currently under investigation. Using an intermittent administration of uridine, leukopenia induced by 5FU (Van Groeningen *et al.*, 1986*b*) could be reversed but thrombocytopenia could not. However, from these preliminary results it cannot be concluded that the clinical application of 5FU in combination with uridine will be successful. In addition, in a randomized study it has to be proven whether in patients the response rate of 5FU will not decrease when 5FU is combined with uridine. From our murine data with Colon 38 it appears that sensitivity of 5FU at a low therapeutic dose is not affected. From our *in vitro* data it may be concluded that low levels of uridine (as observed with oral uridine) might be sufficient to affect the toxicity of 5FU by interference with the incorporation of 5FU into RNA. So the use of higher doses of 5FU in combination with either intermittent i.v. or oral uridine might have improved therapeutic efficacy.

This work was supported by the Netherlands Cancer Foundation 'Koningin Wilhelmina Fonds' by grant IKA 83-16. We thank Mrs E. van Rossum from the Department of Haematology for her help with the analysis of blood samples, and the Clinical Animal Laboratory (Head; B. v.d. Water) for providing facilities for animal experiments. Dr G.J. Peters is a recipient of a senior research fellowship of the Royal Netherlands Academy of Arts and Sciences (KNAW).

### References

AU, J.L.S., BRAMER, S.L. & WIENTJES, M.G. (1987). Pharmacokinetic interaction of 5′-deoxy-5-fluorouridine and uridine in rats. *Proc. Am. Assoc. Cancer Res.*, **28**, 325. (*Abstract 1289*).

CHABNER, B.A. (1982). Pyrimidine antagonists. In *Pharmacological Principles of Cancer Treatment*, Chabner, B.A. (ed) p. 183. W.B. Saunders: Philadelphia.

CIHAK, A. & RADA, B. (1976). Uridine kinase properties, biological significance and chemotherapeutic aspects. *Neoplasma*, **23**, 233.

CORBETT, T.H., GRISWOLD, D.P., ROBERTS, B.J., PECKMAN, J.C. & SCHABEL, F.M. (1977). Evaluation of single agents and combinations of chemotherapeutic agents in mouse colon carcinoma. *Cancer*, **40**, 2660.

CRADOCK, J.C., VISHNUVAJJALA, B.R., CHIN, T.F., HOCHSTEIN, H.D. & ACKERMAN, T.K. (1986). Uridine-induced hyperthermia in the rabbit. *J. Pharm. Pharmacol.*, **38**, 226.

DARNOWSKI, J.W. & HANDSCHUMACHER, R.E. (1986). Tissue uridine pools: Evidence *in vivo* of a concentrative mechanism for uridine uptake. *Cancer Res.*, **46**, 3490.

HOUGHTON, J.A., HOUGHTON, P.J. & WOOTEN, R.S. (1979). Mechanism of induction of gastrointestinal toxicity in the mouse by 5-fluorouracil, 5-fluorouridine and 5-fluoro-2′deoxyuridine. *Cancer Res.*, **39**, 2406.

KARLE, J.N., ANDERSEN, L.W. & CYSYK, R.L. (1984). Effect of plasma concentrations of uridine on pyrimidine bio-synthesis in cultured L1210 cells. *J. Biol. Chem.*, **259**, 67.

KLUBES, P., CERNA, I. & MELDON, M.A. (1982). Uridine rescue from the lethal toxicity of 5-fluorouracil in mice. *Cancer Chemother. Pharmacol.*, **8**, 17.

KLUBES, P.K. & CERNA, I. (1983). Use of uridine rescue to enhance the antitumor selectivity of 5-fluorouracil. *Cancer Res.*, **43**, 3182.

KLUBES, P., GEFFEN, D.B. & CYSYK, R.L. (1986). Comparison of the bio-availability of uridine in mice after either oral or parenteral administration. *Cancer Chemother. Pharmacol.*, **17**, 236.

LEYVA, A., VAN GROENINGEN, C.J., KRAAL, I. & 4 others (1984). Phase I and pharmacokinetic studies of high-dose uridine intended for rescue from 5-fluorouracil toxicity. *Cancer Res.*, **44**, 5928.

MARTIN, D.S., STOLFI, R.L., SAWYER, R.C., SPIEGELMAN, S. & YOUNG, C.W. (1982). High-dose 5-fluorouracil with delayed uridine 'rescue' in mice. *Cancer Res.*, **42**, 3964.

MARTIN, D.S. (1987). Biochemical modulation-perspectives and objectives. In *Proc. 8th Bristol-Myers Symp. on Cancer Res.; New Avenues in Developmental Cancer Chemotherapy*, Harrap, K.R. & Connors, T.A. (eds) p. 113. Academic Press: London.

MOYER, J.D., OLIVER, J.T. & HANDSCHUMACHER, R.E. (1981). Salvage of circulating pyrimidine nucleosides in the rat. *Cancer Res.*, **41**, 3010.

PETERS, G.J., OOSTERHOF, A. & VEERKAMP, J.H. (1983). Pyrimidine metabolism in peripheral and phytohemagglutinin-stimulated mammalian lymphocytes. *Int. J. Biochem.*, **15**, 51.

PETERS, G.J., LAURENSSE, E., LEYVA, A., LANKELMA, J. & PINEDO, H.M. (1986). Sensitivity of human, murine, and rat cells to 5-fluorouracil and 5'deoxy-5-fluorouridine in relation to drug-metabolizing enzymes. *Cancer Res.*, **46**, 20.

PETERS, G.J., VAN GROENINGEN, C.J., LAURENSSE, E. & 4 others (1987*a*). Effect of pyrimidine nucleosides on body temperature of man and rabbit in relation to pharmacokinetic data. *Pharm. Res.*, **4**, 113.

PETERS, G.J., VAN GROENINGEN, C.J., LAURENSSE, E., LANKELMA, J., LEYVA, A. & PINEDO, H.M. (1987*b*). Uridine-induced hypothermia in mice and rats in relation to plasma and tissue levels of uridine and its metabolites. *Cancer Chemother. Pharmacol.*, **20**, 101.

PETERS, G.J., LAURENSSE, E., LEYVA, A. & PINEDO, H.M. (1987*c*). Purine nucleosides as cell-specific modulators of 5-fluorouracil metabolism and cytotoxicity. *Eur. J. Cancer Clin. Oncol.*, **23**, 1869.

PETERS, G.J., VAN DIJK, J., NADAL, J., VAN GROENINGEN, C.J., LANKELMA, J. & PINEDO, H.M. (1987*d*). Diurnal variation in the therapeutic efficacy of 5-fluorouracil against murine colon cancer. *In Vivo*, **1**, 113.

SAWYER, R.C., STOLFI, R.L., SPIEGELMAN, S. & MARTIN, D.S. (1984). Effect of uridine on the metabolism of 5-fluorouracil in the $CD_8F_1$ murine mammary carcinoma system. *Pharm. Res.*, **2**, 69.

SCHOFIELD, R. (1986). Assessment of cytotoxic injury to bone marrow. *Br. J. Cancer*, **53** (Suppl. VII), 115.

VAN GROENINGEN, C.J., LEYVA, A., KRAAL, I., PETERS, G.J. & PINEDO, H.M. (1986*a*). Clinical and pharmacokinetic study of prolonged administration of high-dose uridine intended for rescue from 5-fluorouracil toxicity. *Cancer Treat. Rep.*, **70**, 745.

VAN GROENINGEN, C.J., LEYVA, A., PETERS, G.J., LAURENSSE, E. & PINEDO, H.M. (1986*b*). Reversal of 5-fluorouracil (5-FU) induced myelosuppression by high dose uridine (UR). *Proc. Am Ass. Cancer Res.*, **27**, 169. (*Abstract 670*).

VAN GROENINGEN, C.J., PETERS, G.J., NADAL, J., LEYVA, A., GALL, H. & PINEDO, H.M. (1987). Phase I clinical and pharmacokinetic study of orally administered uridine. *Proc. Am. Ass. Cancer Res.*, **28**, 195. (*Abstract 775*).

VAN KRANENBURG-VOOGD, P.J., KEIZER, H.J. & VAN PUTTEN, L.M. (1978). Experimental chemotherapy of transplantable mouse colon tumors. *Eur. J. Cancer*, **14** (Suppl.) 153.

YEAGER, A.M., LEVIN, J. & LEVIN, F.C. (1983). The effects of 5-fluorouracil on hematopoiesis in studies of murine megakaryocyte CFC, granulocyte macrophage CFC and peripheral blood levels. *Exp. Hematol.*, **11**, 944.